# Mediational role of metabolic syndrome between physical activity, sedentary behavior and non-alcoholic fatty liver disease: a cross-sectional study

**DOI:** 10.1186/s12889-025-22925-8

**Published:** 2025-05-06

**Authors:** Zhaoyu Ren, Hongxuan Fan, Yaya Xue, Xinyu Yang, Xuchang Liu, Jing Luo, Jianqi Zhao, Leigang Wang, Yao Zhang, Bin Liang

**Affiliations:** 1 Department of Cardiology, The Second Hospital of Shanxi Medical University, Wuyi Road, Taiyuan, Shanxi 030000 China; 2 Beijing Tsinghua Changgung Hospital, School of Clinical Medicine, Tsinghua University, Beijing, China; 3 The First Hospital of Shanxi Medical University, Taiyuan, Shanxi China

**Keywords:** Sedentary behavior, Physical activity, Recreational activity, Metabolic syndrome, Non-alcoholic fatty liver disease, Mediation analysis

## Abstract

**Background:**

Physical activity (PA), sedentary behavior (SB), metabolic syndrome (MetS), and non-alcoholic fatty liver disease (NAFLD) have been linked in previous studies. Nevertheless, it is unclear whether MetS has a mediating influence on the relationships among physical activity, sedentary behavior, and non-alcoholic fatty liver disease. This study aims to assess the connections between physical activity, sedentary behavior, and non-alcoholic fatty liver disease and to explore the extent to which metabolic syndrome acts as a mediator in this association.

**Methods:**

A total of 3351 adults from the National Health and Nutrition Examination Survey (NHANES) from 2017 to 2018 were included in our study. Physical activity and sedentary behavior were categorized as work activity (WA), recreational activity (RA), walking/bicycling (for commuting) and sedentary behavior to investigate the association with metabolic syndrome and non-alcoholic fatty liver disease. Besides, mediation analysis was utilized to determine the extent to which metabolic syndrome mediates the relationships among inadequate physical activity, prolonged sedentary behavior, and non-alcoholic fatty liver disease.

**Results:**

Regression analysis revealed that a reduced risk of developing NAFLD was associated with sufficient recreational activity (OR = 0.61, 95% CI: 0.44–0.83, *P* = 0.004), while an increased risk of MetS was observed in sedentary behavior group (OR = 1.28, 95% CI: 1.00–1.64, *P* < 0.05). In addition, strong associations were detected between MetS and NAFLD. Mediation analysis indicated that metabolic syndrome accounts for 17.9% of the influence that recreational activity has on the risk of NAFLD. Subgroup analysis indicated sex differences in these associations. Specifically, recreational activity may not significantly influence the risk of developing NAFLD in females, and the mediating role of MetS was no longer significant in both sex-specific subgroups.

**Conclusion:**

In the general adult population, metabolic syndrome may account for nearly 18% of the association between insufficient recreational activity and non-alcoholic fatty liver disease.

**Supplementary Information:**

The online version contains supplementary material available at 10.1186/s12889-025-22925-8.

## Introduction

A significant proportion of individuals (28.6%−31.1%) suffer from non-alcoholic fatty liver disease (NAFLD), which is the primary cause of chronic liver disease and a critical global health issue [1]. The global burden of NAFLD is escalating due to the increasing rates of obesity, with the prevalence of NAFLD reaching 69.99% (95% CI 65.40–74.21) among overweight individuals [2]. NAFLD is becoming a leading cause of end-stage liver disease worldwide and is recognized as an etiological factor for hepatocellular carcinoma (HCC), even in the absence of underlying cirrhosis [3]. This contributes to higher mortality rates, and the trend is expected to continue to rise sharply [4].

To assess liver fibrosis and steatosis, transient elastography (TE), commonly known as FibroScan®, is a noninvasive technique with acceptable accuracy and reproducibility. Parameters such as the controlled attenuation parameter (CAP) and liver stiffness measurement (LSM) obtained through TE have been established as independent predictors of all-cause mortality in individuals with NAFLD and type 2 diabetes [5] and in the U.S. adult population [6].

Metabolic syndrome (MetS), also known as"syndrome X"or"insulin resistance syndrome", comprises a cluster of clinical syndromes that significantly impact overall health. It is characterized by a combination of factors, including obesity, particularly abdominal obesity, hyperglycemia (which can manifest as diabetes mellitus or impaired glucose regulation), and dyslipidemia, involving either hypertriglyceridemia or hypo-high-density lipoprotein cholesterolemia. According to a nationwide study, the weighted prevalence of metabolic syndrome in the U.S. stands at 34.7% (95% CI, 33.1%−36.3%), and this prevalence continues to rise annually [7].

The etiology of NAFLD is multifactorial, encompassing disorders of lipid metabolism, nutritional causes, and the influence of certain medications, with MetS being recognized as a central component [8, 9]. When associated with MetS, it’s called primary NAFLD; otherwise, it’s secondary NAFLD. Recent research [10, 11] has shown a correlation between the number of metabolic components present in individuals with NAFLD and an increased risk of mortality. These findings underscore the importance of metabolic variables in the development of NAFLD [12]. Consequently, the concept of"Metabolic Dysfunction-Associated Fatty Liver Disease"(MAFLD) has been increasingly embraced by the medical community. This newer term takes into account the established metabolic risk factors alongside the traditional aspects of NAFLD [13].

There is abundant evidence that sedentary lifestyles are associated with a greater incidence of metabolic syndrome and non-alcoholic fatty liver disease [14, 15]. Exercise offers evident health benefits to individuals with obesity, including the potential for weight loss and improved insulin sensitivity [16]. In the U.S. population, increased physical activity was linked to a reduced risk of NAFLD [17]. A meta-analysis of randomized controlled trials demonstrated that individuals with non-alcoholic fatty liver disease can experience metabolic improvements by making changes to their sedentary habits [18]. Nevertheless, based on a nationally representative U.S. population survey from 2001 to 2016, the estimated prevalence of sedentary behaviors has typically remained high. All age groups have witnessed an increase in both leisure-time computer usage and total sitting time [19]. Consequently, the modification of sedentary behavior poses a challenging yet essential task.

To further investigate the relationship between physical activity (PA), sedentary behavior (SB), MetS, and NAFLD, we conducted a mediation analysis to assess the indirect role of MetS and identify potential methods for mitigating the disease burden of NAFLD.

## Methods

### Patient information

**Fig. 1:**
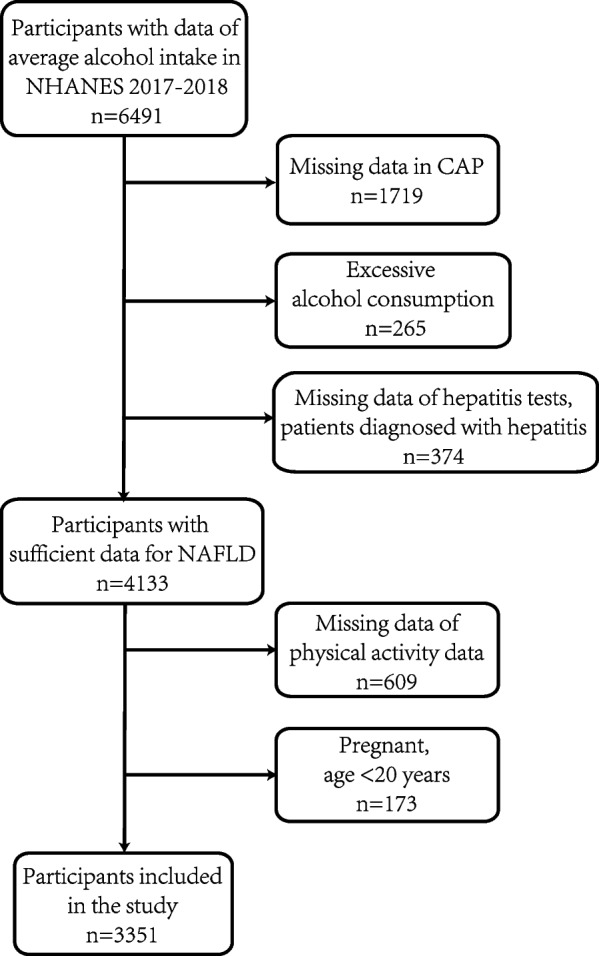
Flowchart of selection for NAFLD in NHANES 2017–2018. Note: NAFLD, non-alcoholic fatty liver disease; CAP, controlled-attenuation parameter

The individuals included in this study were collected from the National Health and Nutrition Examination Surveys (NHANES) 2017–2018 database. NHANES is a continuous cross-sectional study designed to monitor the health of the American population at the national level. Participants are selected from the non-institutionalized, general population of the United States via a stratified and multi-stage probability cluster selection approach, and in-home interviews and mobile examinations were conducted. Written informed consent was provided by all survey participants, and ethical approval was granted by the Ethics Review Board of the National Center for Health Statistics. Participants were excluded for missing data, pregnancy, or being under 20 years old, as shown in Fig. 1.

### Definition and assessment of non-alcoholic fatty liver disease

Controlled attenuation parameter (CAP), obtained by transient elastography (FibroScan®), is considered optimal for reflecting hepatic steatosis, with a cutoff value of ≥ 285 dB/m (sensitivity of 80% and specificity of 77%) [20]. Alcohol intake daily is the average amount of alcohol consumed per day, measured in drinks, from dietary data collected over two days. One drink is defined as the amount of alcoholic beverage containing 0.6 fluid ounces or 14 g of alcohol. Only participants who consumed less than three or two standard drinks per day on average, for males and females, respectively, were included in the present analysis. Additionally, individuals who had been diagnosed with viral hepatitis B and C were disqualified [21].

### Assessment of physical activity and sedentary behavior

The Physical Activity Questionnaire, which is based on the Global Physical Activity Questionnaire [22], was utilized as an effective tool for collecting physical activity data. Detailed information about the questionnaire items can be found in the Table S1. According to the Guidelines of the World Health Organization (WHO), we defined sufficient physical activity for participants who completed at least 150 min per week of moderate-intensity physical activity (MPA), 75 min per week of vigorous-intensity physical activity (VPA), or an equivalent combination of both (VPA of 1 min is equal to MPA of 2 min) [23]. Based on the reported number of days and time spent (in minutes) on moderate or vigorous work activity, we calculated the weekly time spent on vigorous work activity (VWA), moderate work activity (MWA), and moderate-to-vigorous work activity (MVWA), with MVWA determined by the formula MVWA = MWA + 2 VWA. Participants of the MVWA group were classified as having either insufficient MVWA (< 150 min/week) or sufficient MVWA (≥ 150 min/week). The calculation of vigorous recreational activity (VRA), moderate recreational activity (MRA), and moderate-to-vigorous recreational activity (MVRA) follows the same approach as work activity. Additionally, MVRA was also divided into sufficient (≥ 150 min/week) and insufficient (< 150 min/week) groups. Walking/Bicycling (for commuting) time was based on self-reported days and time spent traveling to and from places by walking or using a bicycle. The participants in the Walking/Bicycling group were classified as having sufficient activity if they had engaged in these activities for at least 150 min per week. Additionally, sedentary time was based on the self-reported amount of sitting time during a typical day. The participants were classified as having sedentary behavior if they reported sitting for 480 min or more per day [24]. (see Table 1).

**Table 1:** Definition of main physical activity terms used in this study

Physical activity	Term	Definition
Work activity	VWA	vigorous work activity
	MWA	moderate work activity
	MVWA	moderate-to-vigorous work activity
	MVWA group	insufficient (MVWA < 150 min/week)
		sufficient (MVWA ≥ 150 min/week)
Recreational activity	VRA	vigorous recreational activity
	MRA	moderate recreational activity
	MVRA	moderate-to-vigorous recreational activity
	MVRA group	insufficient (MVRA < 150 min/week)
		sufficient (MVRA ≥ 150 min/week)
Walking/Bicycling	Walking/Bicycling group	insufficient (< 150 min/week)
		sufficient (≥ 150 min/week)
Sedentary behavior	Sedentary behavior group	insufficient (< 480 min/day)
		sufficient (≥ 480 min/day)

### Definition of metabolic syndrome

According to the diagnostic criteria for metabolic syndrome (MetS) proposed by the International Diabetes Federation criteria in 2009 [25], participants were classified as having MetS if they met three or more of the following criteria: Elevated waist circumference (≥ 94 cm for males [≥ 90 cm for Asian or Mexican males], ≥ 80 cm for females), Elevated triglycerides (≥ 1.7 mmol/L, or drug treatment for elevated triglycerides), Reduced HDL-C (< 1.0 mmol/L in males, < 1.3 mmol/L in females, or drug treatment for reduced HDL-C), Elevated blood pressure (systolic ≥ 130 mmHg and/or diastolic ≥ 85 mmHg, current use of antihypertensive medication, or a previous diagnosis of hypertension), Elevated fasting glucose (≥ 100 mg/dL, current use of glucose-lowering medication, or a previous diagnosis of diabetes). Physical data, such as height, weight, waist circumference, and body mass index (BMI), were also collected. Blood pressure was measured three times after the patient rested in a seated position for five minutes, and the mean of the three readings was used for analysis. Detailed information about the laboratory instruments and measuring equipment used can be found on the official website.

### Statistical analysis

The complex questionnaires used in the NHANES rendered the application of standard estimates inappropriate. Therefore, all analyses had to be properly weighted to align with the United States population. Weighted estimates were calculated following the analytical guidelines provided online by the NHANES for the years of 2017–2018. Data analysis was performed using Empower Statistics version 4.2, R (version 4.2.2), and the nhanesR package (version 0.9.5.0). Continuous variables were analyzed for survey-weighted means with 95% confidence intervals (CI) using t-tests, one-way ANOVA tests, and Wilcoxon rank-sum tests, whereas categorical variables were assessed for frequencies and survey-weighted percentages through survey-weighted Chi-square tests. Multivariate logistic regression analysis or linear regression analysis was employed to investigate potential predictors of NAFLD and identify independent predictors. A subgroup analysis was conducted to examine the relationship between activity patterns (physical activity or sedentary behavior) and NAFLD across different sexes. Interaction analysis and mediation analysis were used to assess the impact of MetS on sedentary behavior or physical activity leading to NAFLD. In mediation analysis, the direct effect refers to the association between physical activity/sedentary behavior and NAFLD, while the indirect effect represents the portion of this association that is mediated through MetS. The proportion mediated indicates the percentage of the total effect that is attributable to the mediating role of MetS. This mediation analysis provides evidence to elucidate the underlying mechanisms by which physical activity or sedentary behavior may contribute to the development of NAFLD. In this study, sex, age, race, education, BMI, marital status, family poverty income ratio, alcohol intake and smoking status were considered potential confounders. It was considered statistically significant if *p*-value < 0.05.

## Results

### Demographic characteristics and clinical laboratory data of NAFLD and non-NAFLD

Figure 1 displays the flowchart of selection for NAFLD in NHANES 2017–2018 and Table 2 shows the characteristics of the participants. A total of 3,351 individuals were included in the study, of whom 1,340 had NAFLD. Among the NAFLD patients, 46.56% were female, and 53.44% were male. In the non-NAFLD group (*N* = 2011), 43.77% were male, and 56.23% were female. The sex difference was statistically significant (*P* < 0.05). Additionally, factors such as age, MVRA, BMI, race, education level, marital status, and smoking status were significantly different between the groups (*P* < 0.05). The prevalence of different diseases was significantly higher in the NAFLD group: hypertension (46.97% vs. 22.72%), diabetes (28.74% vs. 7.53%), and MetS (61.44% vs. 24.52%). Table 3 compared the laboratory data between the NAFLD and non-NAFLD groups, as well as the laboratory parameters across different sexes. Among these markers related to liver function, alanine aminotransferase (ALT), alkaline phosphatase (ALP), aspartate aminotransferase (AST), gamma-glutamyl transferase (GGT), globulin, and triglycerides levels were elevated in patients with NAFLD, while total bilirubin and albumin levels were decreased. While all these markers showed statistically significant differences, only triglycerides levels in the NAFLD group exceeded the normal range; the other markers, despite the differences, remained within normal limits. Furthermore, the levels of glucose, osmolality, blood urea nitrogen, and uric acid in the frozen serum of patients increased, whereas the levels of iron and chloride in the serum decreased, with statistical significance (*P* < 0.05). Additionally, these markers remained within the normal range in both groups.

**Table 2:** Demographic characteristics of NAFLD and Non-NAFLD

	Total	Non-NAFLD	NAFLD	*P*-value
	(*N* = 3351)	(*N* = 2011)	(*N* = 1340)	
Age	48.30(46.75,49.86)	46.08(44.28,47.87)	52.05(50.49,53.62)	**< 0.0001**
Sex				**0.01**
Female	1778(52.63)	1153(56.23)	625(46.56)	
Male	1573(47.37)	858(43.77)	715(53.44)	
Race				**0.01**
Non-Hispanic White	1215(62.64)	702(63.39)	513(61.37)	
Non-Hispanic Black	789(11.07)	539(12.44)	250(8.76)	
Non-Hispanic Asian	418(5.87)	272(6.28)	146(5.19)	
Other Hispanic	318(6.85)	195(6.85)	123(6.84)	
Mexican American	449(9.33)	203(7.02)	246(13.23)	
Other Race	162(4.24)	100(4.02)	62(4.61)	
Marital status				**0.001**
Married	1729(55.50)	958(52.09)	771(61.28)	
Never married	601(19.46)	417(22.94)	184(13.62)	
Divorced	377(9.92)	222(10.33)	155(9.24)	
Widowed	247(5.45)	152(5.41)	95(5.51)	
Separated	117(2.60)	74(2.61)	43(2.59)	
Living with partner	278(7.04)	186(6.62)	92(7.76)	
Education level				**0.01**
Less than 9 th grade	239(3.11)	138(2.99)	101(3.31)	
9-11 th grade (Includes 12 th grade with no diploma)	334(6.40)	197(5.85)	137(7.34)	
High school graduate/GED or equivalent	776(26.74)	454(25.63)	322(28.64)	
Some college or AA degree	1131(30.83)	656(28.98)	475(33.97)	
College graduate or above	866(32.89)	564(36.56)	302(26.74)	
BMI	29.93(29.35,30.50)	27.35(26.82,27.88)	34.26(33.51,35.02)	**< 0.0001**
Family income to poverty	3.12(2.99,3.25)	3.16(3.00,3.32)	3.06(2.88,3.23)	0.4
Alcohol intake daily	0.26(0.23,0.29)	0.27(0.23,0.31)	0.24(0.18,0.29)	0.39
MetS				** < 0.0001**
no	1922(61.74)	1439(75.48)	483(38.56)	
yes	1429(38.26)	572(24.52)	857(61.44)	
Diabetes				**< 0.0001**
no	2614(84.57)	1737(92.47)	877(71.26)	
yes	737(15.43)	274(7.53)	463(28.74)	
Hypertension				**< 0.0001**
no	2065(68.11)	1386(77.28)	679(53.03)	
yes	1280(31.71)	620(22.72)	660(46.97)	
smoke				**0.01**
never	1987(60.55)	1242(63.17)	745(56.15)	
former	830(25.18)	432(22.18)	398(30.25)	
now	534(14.26)	337(14.66)	197(13.60)	
VWA (mins/week)	229.19(189.93,268.45)	245.46(203.79,287.13)	201.76(150.34,253.17)	0.09
MWA(mins/week)	445.16(375.04,515.28)	440.12(378.26,501.98)	453.66(337.52,569.80)	0.8
MVWA (mins/week)	903.54(782.58,1024.51)	931.04(813.24,1048.84)	857.17(681.24,1033.10)	0.36
MVWA group				0.12
Insufficient	2006(54.54)	1219(56.39)	787(51.44)	
Sufficient	1345(45.46)	792(43.61)	553(48.56)	
VRA (mins/week)	83.55(64.68,102.41)	103.30(79.47,127.14)	50.23(39.42, 61.04)	**< 0.0001**
MRA (mins/week)	104.95(86.30,123.61)	102.50(89.07,115.93)	109.08(66.38,151.79)	0.77
MVRA (mins/week)	272.05(231.13,312.97)	309.11(258.01,360.21)	209.55(159.48,259.61)	**0.01**
MVRA group				**< 0.0001**
Insufficient	2230(59.25)	1246(52.78)	984(70.15)	
Sufficient	1121(40.75)	765(47.22)	356(29.85)	
Walking/Bicycling time (mins/week)	76.28(48.69,103.87)	82.81(40.77,124.85)	65.28(43.84, 86.71)	0.48
Walking/Bicycling group				0.88
Insufficient	2943(89.12)	1749(89.20)	1194(88.98)	
Sufficient	408(10.88)	262(10.80)	146(11.02)	
Sedentary time(mins/day)	405.51(350.01,461.00)	411.65(327.52,495.78)	395.16(334.46,455.87)	0.77
sedentary behavior group				0.5
no	2385(69.03)	1465(69.76)	920(67.78)	
yes	966(30.97)	546(30.24)	420(32.22)	

Boldface indicates statistical significance

Continuous variables are shown as survey-weighted means (95% CI), and categorical variables are presented as frequencies (survey-weighted percentages), *VWA* vigorous work activity, *MWA* moderate work activity, *MVWA* moderate-to-vigorous work activity, *VRA* vigorous recreational activity, *MRA* moderate recreational activity, *MVRA* moderate-to-vigorous recreational activity

**Table 3:** Laboratory data for NAFLD and non-NAFLD groups by sex

	Total	Non-NAFLD (*N* = 2011)			NAFLD (*N* = 1340)			*P*-value	Normal Reference Range
	(*N* = 3351)	Total	Female	Male	Total	Female	Male		
Alanine Aminotransferase (ALT) (IU/L)	22.41(21.65,23.17)	19.61(18.69,20.54)	16.86(15.62,18.10)	23.11(21.65,24.56)	27.11(25.74,28.47)	22.46(20.79,24.13)	31.15(29.40,32.90)	**< 0.0001**	7–56
Aspartate Aminotransferase (AST) (IU/L)	21.59(20.82,22.36)	20.96(19.93,21.98)	19.18(18.53,19.82)	23.22(21.13,25.30)	22.65(21.78,23.52)	21.19(20.01,22.37)	23.92(22.84,24.99)	**0.02**	10–40
Total Protein (g/L)	71.05(70.69,71.41)	70.94(70.58,71.31)	70.66(70.11,71.22)	71.30(70.93,71.67)	71.23(70.84,71.63)	70.95(70.46,71.44)	71.48(70.99,71.96)	**0.05**	60–83
Albumin, refrigerated serum (g/L)	41.12(40.81,41.42)	41.32(40.97,41.67)	40.59(40.12,41.05)	42.26(41.91,42.60)	40.77(40.38,41.16)	39.79(39.35,40.23)	41.62(41.14,42.11)	**0.02**	35–50
Globulin (g/L)	29.93(29.62,30.25)	29.62(29.23,30.01)	30.08(29.65,30.50)	29.04(28.65,29.44)	30.46(30.10,30.82)	31.16(30.73,31.59)	29.85(29.26,30.45)	**0.001**	10–48
Total Bilirubin (umol/L)	8.06(7.77,8.34)	8.28(7.89,8.67)	6.85(6.52,7.17)	10.10(9.43,10.77)	7.68(7.23,8.12)	6.53(6.02,7.03)	8.67(8.18, 9.17)	0.07	1.7–20.5
Gamma Glutamyl Transferase (GGT) (IU/L)	27.14(26.02,28.25)	22.87(21.35,24.40)	19.50(17.04,21.96)	27.16(24.25,30.07)	34.28(32.48,36.09)	28.55(26.29,30.80)	39.27(36.52,42.03)	**< 0.0001**	8–61 (Male) 5–36 (Female)
Alkaline Phosphatase (ALP) (U/L)	76.43(74.73,78.13)	73.57(71.30,75.84)	73.97(71.03,76.90)	73.07(70.06,76.08)	81.22(79.01,83.43)	85.12(82.43,87.82)	77.83(74.24,81.42)	**< 0.001**	40–129 (Male) 35–104(Female)
Bicarbonate (mmol/L)	25.67(25.30,26.05)	25.73(25.35,26.11)	25.34(24.97,25.70)	26.23(25.73,26.72)	25.58(25.16,26.00)	25.47(25.01,25.94)	25.67(25.18,26.16)	0.26	22–29
Blood Urea Nitrogen (mmol/L)	5.34(5.20,5.47)	5.24(5.11,5.36)	5.04(4.89,5.19)	5.49(5.33,5.64)	5.51(5.31,5.70)	5.17(4.94,5.40)	5.80(5.56,6.04)	**0.01**	2.5–7.1
Creatinine, refrigerated serum (umol/L)	78.08(76.55,79.60)	77.30(76.47,78.14)	68.00(66.83,69.17)	89.10(87.66,90.55)	79.37(76.16,82.58)	66.28(64.31,68.25)	90.77(86.25,95.28)	0.18	53–106 (Male) 44–97 (Female)
Uric acid (umol/L)	318.86(314.79,322.93)	304.25(299.49,309.01)	272.28(266.65,277.91)	344.77(336.68,352.85)	343.36(337.38,349.33)	310.76(303.12,318.40)	371.72(362.02,381.42)	**< 0.0001**	202–416 (Male) 143–357(Female)
Glucose, refrigerated serum (mmol/L)	5.56(5.46,5.65)	5.16(5.10,5.22)	5.08(5.00,5.16)	5.26(5.17,5.34)	6.22(6.04,6.40)	6.15(5.76,6.54)	6.28(6.02,6.55)	**< 0.0001**	3.9–7.8
Lactate Dehydrogenase (LDH) (U/L)	156.63(153.96,159.30)	155.05(151.68,158.42)	155.77(152.10,159.43)	154.12(149.77,158.48)	159.31(155.50,163.11)	161.12(156.87,165.38)	157.71(153.35,162.06)	0.1	125–243
Osmolality (mmol/kg)	280.62(279.04,282.20)	280.11(278.71,281.52)	279.57(277.95,281.20)	280.79(279.64,281.95)	281.47(279.55,283.40)	280.78(278.74,282.81)	282.08(280.17,283.98)	**0.002**	275–295
Potassium (mmol/L)	4.08(4.02,4.14)	4.07(4.01,4.14)	4.01(3.95,4.08)	4.15(4.08,4.23)	4.09(4.02,4.16)	4.05(3.97,4.13)	4.12(4.04,4.20)	0.59	3.5–5.0
Sodium (mmol/L)	140.18(139.31,141.05)	140.17(139.40,140.94)	140.02(139.14,140.89)	140.36(139.69,141.03)	140.19(139.14,141.25)	140.03(138.93,141.14)	140.33(139.24,141.43)	0.9	135–145
Chloride (mmol/L)	100.83(100.43,101.24)	101.03(100.66,101.39)	101.16(100.71,101.61)	100.86(100.56,101.16)	100.51(99.98,101.04)	100.61(100.09,101.12)	100.43(99.79,101.06)	**0.01**	98–106
Total Calcium (mmol/L)	2.33(2.31,2.34)	2.32(2.31,2.34)	2.32(2.31,2.33)	2.33(2.32,2.35)	2.33(2.32,2.34)	2.32(2.30,2.34)	2.34(2.32,2.35)	0.46	2.12–2.62
Phosphorus (mmol/L)	1.16(1.15,1.17)	1.17(1.15,1.18)	1.19(1.18,1.21)	1.13(1.11,1.15)	1.15(1.14,1.16)	1.17(1.15,1.19)	1.13(1.11,1.15)	0.16	0.81–1.45
Iron, refrigerated serum (umol/L)	15.75(15.29,16.20)	16.11(15.59,16.64)	14.99(14.45,15.53)	17.54(16.60,18.49)	15.14(14.55,15.72)	14.00(13.03,14.97)	16.13(15.58,16.68)	**0.01**	10.7–30.4
Cholesterol, refrigerated serum (mmol/L)	4.91(4.81,5.00)	4.89(4.80,4.98)	4.99(4.86,5.12)	4.76(4.62,4.89)	4.94(4.81,5.07)	5.11(4.96,5.25)	4.79(4.63,4.96)	0.39	< 5.18
Triglycerides, refrigerated serum (mmol/L)	1.66(1.55,1.76)	1.39(1.31,1.47)	1.28(1.21,1.35)	1.53(1.37,1.69)	2.10(1.97,2.23)*	1.94(1.81,2.06)*	2.24(2.01,2.47)*	**< 0.0001**	< 1.7

^*^indicators that are out of the normal reference range

### Regression analysis of physical activity, sedentary behavior and NAFLD

Table 4 displays the effects of four different physical activity patterns on the risk of developing NAFLD in the general population. It appears that the duration of work activity, time spent walking or bicycling (for commuting), and sedentary time are not significantly associated with NAFLD. Only recreational activity was significantly associated with NAFLD. Logistic regression analysis revealed that the duration of vigorous recreational activity was associated with NAFLD (OR = 0.9987, 95% CI = 0.9979–0.9996, *p* = 0.0058). In the context of the recreational activity group, the sufficient MVRA group meeting the WHO standards had a lower risk for NAFLD (OR = 0.61, 95% CI = 0.44–0.83, *p* = 0.004). In addition, linear regression analysis indicated that sufficient MVRA significantly reduced the CAP index (β = −13.97, 95% CI = −19.53—−8.40, *p* < 0.0001).

**Table 4:** Regression analysis of physical activity and NAFLD

		OR(95% CI)	*P*-value	β(95% CI)	*P*-value
Work activity	VWA (mins/week)	0.9999(0.9996,1.0001)	0.2661	−0.0018(−0.0050, 0.0014)	0.2506
	MWA (mins/week)	1.0001(0.9998,1.0003)	0.446	0.0014(−0.0002, 0.0030)	0.0903
	MVWA (mins/week)	0.99999(0.99994,1.00005)	0.81403	−0.0002(−0.0010, 0.0006)	0.5893
	MVWA group	1.26(0.84,1.90)	0.24	4.46(−1.49, 10.40)	0.13
Recreational activity	VRA (mins/week)	0.9987(0.9979,0.9996)	0.0058	−0.0018(−0.0054, 0.0018)	0.3096
	MRA (mins/week)	1.0002(0.9993,1.0012)	0.6034	0.0024(−0.0130, 0.0177)	0.7481
	MVRA (mins/week)	0.9997(0.9993,1.0001)	0.1554	−0.0008(−0.0025, 0.0009)	0.3212
	MVRA group	0.61(0.44,0.83)	0.004	−13.97(−19.53, −8.40)	< 0.0001
Walking/Bicycling	Walking/Bicycling time (mins/week)	0.99996(0.99981,1.00012	0.62146	−0.0009(−0.0031, 0.0013)	0.4018
	Walking/Bicycling group	1.25(0.70,2.22)	0.43	−0.51(−15.10, 14.07)	0.94
Sedentary behavior	Sedentary time (mins/day)	0.99998(0.99972,1.00023)	0.85615	−0.0007(−0.0050, 0.0037)	0.749
	sedentary behavior group	1.09(0.68,1.73)	0.71	0.79(−9.74, 11.31)	0.88

*OR* effect value when outcomes are categorical variables, *CI* confidence interval, *β* effect value when outcomes are continuous variables. Adjusted for sex, age, race, education, marital status, BMI, family income to poverty, alcohol intake daily and smoking. *VWA* vigorous work activity, *MWA* moderate work activity, *MVWA* moderate-to-vigorous work activity, *VRA* vigorous recreational activity, *MRA* moderate recreational activity, *MVRA* moderate-to-vigorous recreational activity

### Subgroup analysis of physical activity, sedentary behavior and NAFLD stratified by sex

Table 5 displays the subgroup analysis of the associations between four distinct physical activity patterns and NAFLD, stratified by sex. Regardless of sex, work activity and sedentary behavior are not significantly associated with NAFLD. In the recreational activity group, sufficient MVRA was found to have a positive effect on reducing the risk of NAFLD in males and lowering the CAP index in females. Compared with insufficient MVRA, sufficient MVRA decreased the risk of NAFLD in males (OR = 0.44, 95% CI = 0.28–0.69, *p* = 0.002). Similarly, for females, sufficient MVRA also decreased the CAP index (β = −10.82, 95% CI = −18.85—−2.80, *p* = 0.01). These findings indicate that MVRA has a stronger protective effect on NAFLD. In the Walking/bicycling group, the results showed that sufficient walking/bicycling time lowered the CAP index (β = −16.04, 95% CI = −23.48 – −8.59, *p* < 0.001).

**Table 5:** Subgroup analysis between physical activity and NAFLD stratified by sex

	Sex	OR (95% CI)	*P*-value	β **(95% CI)**	*P*-value
MVWA group	male	0.91(0.59, 1.42)	0.67	3.38(−6.17, 12.93)	0.46
	female	1.76(0.98,3.16	0.06	6.03(−1.88, 13.94)	0.12
MVRA group	male	0.44(0.28, 0.69)	**0.002**	5.96(−7.98, 19.91)	0.38
	female	0.89(0.50,1.58)	0.68	−10.82(−18.85, −2.80)	**0.01**
Walking/bicycling group	male	1.84(0.92, 3.69)	0.08	−16.04(−23.48, −8.59)	**< 0.001**
	female	0.71(0.29,1.74)	0.42	−10.57(−33.62, 12.48)	0.34
Sedentary behavior group	male	1.24(0.74, 2.07)	0.38	2.73(−8.65, 14.11)	0.62
	female	0.92(0.50,1.69)	0.77	−1.43(−13.90, 11.04)	0.81

Boldface indicates statistical significance

Adjusted for sex, age, race, education, marital status, BMI, family income to poverty, alcohol intake daily and smoking. OR: effect value when outcomes are categorical variables; *CI* confidence interval; β: effect value when outcomes are continuous variables; *MVWA* moderate-to-vigorous work activity, *MVRA* moderate-to-vigorous recreational activity

### Binary logistic regression of MetS with physical activity patterns and NAFLD

Table 6 shows the associations between the four physical activity patterns and MetS. We assessed the relationships between these patterns and MetS via binary logistic regression analysis. Compared with insufficient MVWA, sufficient MVWA did not reduce the risk of metabolic syndrome. Additionally, binary logistic regression analysis indicated that sedentary behavior was associated with a higher risk of developing MetS, with an OR of 1.28 (95% CI: 1.00–1.64, *p* < 0.05).

**Table 6:** Binary logistic regression of four physical activity patterns and MetS

	OR (95% CI)	*P*-value
MVWA group	0.85(0.60,1.21)	0.35
MVRA group	0.87(0.65,1.18)	0.35
Walking/bicycling group	1.29(0.90,1.84)	0.15
Sedentary behavior group	1.28(1.00,1.64)	**< 0.05**

Boldface indicates statistical significance

Adjusted for sex, age, race, education, marital status, BMI, family income to poverty, alcohol intake daily and smoking. *OR* odds ratio, *MVWA* moderate-to-vigorous work activity, *MVRA* moderate-to-vigorous recreational activity

Subsequently, regression analysis was used to investigate the impact of metabolic syndrome on NAFLD. Surprisingly, metabolic syndrome was found to significantly increase the probability of NAFLD (OR = 2.3, 95% CI = 1.78–2.96, *p* < 0.0001) and elevate CAP index (β = 26, 95% CI = 15.94–36.07, *p* < 0.0001) compared with individuals without MetS, as shown in Table 7.

**Table 7:** Regression analysis of MetS and NAFLD

		OR/β	95%CI	*P*-value
Metabolic syndrome	OR	2.3	1.78,2.96	**< 0.0001**
	β	26	15.94, 36.07	**< 0.0001**

Boldface indicates statistical significance

OR: effect value when outcomes are categorical variables; *CI* confidence interval, *β* effect value when outcomes are continuous variables. Adjusted for sex, age, race, education, marital status, BMI, family income to poverty, alcohol intake daily and smoking

### The mediation and subgroup analyses of MetS

**Fig. 2:**
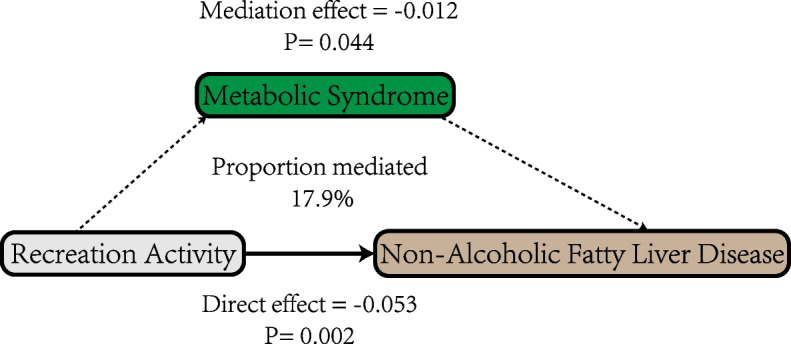
Mediating pathway of MetS between the Recreation activity and NAFLD. Note: Graph of the mediation effect of MetS on the relationship between Recreation activity and NAFLD. The solid line represents the direct effect of recreational activity on NAFLD, while the dashed line indicates the mediating role of MetS between the two. The direct effect demonstrates the direct impact of recreational activity on NAFLD, while the mediation effect illustrates the mediating role of MetS. NAFLD, non-alcoholic fatty liver disease

We examined the interaction of four physical activity patterns with MetS on NAFLD and found that only walking/bicycling group was interacted with MetS, as shown in Table S2. Table 8 presents the total effect, mediation effect, direct effect, and proportion mediated for the impact of the four activity patterns on liver steatosis through MetS. Among the four physical activity patterns, MetS was significantly associated as a mediator only in the relationship between recreational activity and NAFLD, with a mediation proportion of 17.9%, as shown in Fig. 2. However, this mediation effect disappeared in the sex subgroups. To determine whether the four physical activity patterns are associated with NAFLD in MetS patients and non-MetS patients, as shown in Table 9, we conducted subgroup analyses. Among MetS patients, none of the physical activity patterns was associated with NAFLD. However, in patients without MetS, sufficient recreational activity was associated with a lower likelihood of NAFLD, with an OR (95% CI) of 0.55 (0.35, 0.87).

**Table 8:** Mediation analysis of MetS on four physical activity patterns associated with NAFLD with subgroup analysis by sex

	Total		Female		Male	
	OR (95% CI)	*P*-value	OR (95% CI)	*P*-value	OR (95% CI)	*P*-value
**MVWA group**						
Total effect	−0.005 (−0.034, 0.031)	0.904	0.055 (0.006, 0.093)	**0.036**	−0.068 (−0.112, −0.02)	**0.004**
Mediation effect	−0.01 (−0.015, 0.001)	0.09	−0.001 (−0.019, 0.005)	0.204	−0.009 (−0.017, 0.002)	0.146
Direct effect	0.006 (−0.026, 0.037)	0.746	0.056 (0.013, 0.099)	**0.018**	−0.059 (−0.105, −0.014)	**0.012**
Propotion mediated	2.188 (−6.646, 6.73)	0.862	−0.023 (−1.082, 0.128)	0.232	0.131 (−0.051, 0.35)	0.15
**MVRA group**						
Total effect	−0.065 (−0.096, −0.03)	** < 0.0001**	−0.015 (−0.071, 0.026)	0.424	−0.097 (−0.143, −0.051)	**< 0.0001**
Mediation effect	−0.012 (−0.017, 0)	**0.044**	−0.002 (−0.021, 0.004)	0.208	−0.008 (−0.02, 0.001)	0.078
Direct effect	−0.053 (−0.086, −0.022)	**0.002**	−0.012 (−0.06, 0.035)	0.628	−0.089 (−0.135, −0.042)	**< 0.0001**
Propotion mediated	0.179 (0.007, 0.332)	**0.044**	0.162 (−5.638, 4.704)	0.52	0.087 (−0.012, 0.221)	0.078
**Walking/Bicycling group**						
Total effect	−0.022 (−0.068, 0.028)	0.434	−0.041 (−0.122, 0.027)	0.212	0.003 (−0.058, 0.069)	0.828
Mediation effect	−0.007 (−0.014, 0.007)	0.51	−0.006 (−0.027, 0.006)	0.202	−0.0003 (−0.01, 0.013)	0.872
Direct effect	−0.015 (−0.064, 0.03)	0.534	−0.035 (−0.109, 0.039)	0.36	0.003 (−0.06, 0.068)	0.848
Propotion mediated	0.307 (−3.373, 2.263)	0.672	0.156 (−1.531, 2.555)	0.354	−0.101 (−2.209, 2.674)	0.836
**Sedentary behavior group**						
Total effect	0.029 (−0.003, 0.065)	0.066	0.009 (−0.044, 0.052)	0.83	0.063 (0.012, 0.118)	**0.018**
Mediation effect	0.0003 (−0.005, 0.011)	0.452	0.002 (−0.015, 0.009)	0.648	0.007 (−0.003, 0.018)	0.168
Direct effect	0.028 (−0.007, 0.059)	0.102	0.007 (−0.038, 0.054)	0.718	0.056 (0.007, 0.11)	**0.03**
Propotion mediated	0.009 (−0.641, 0.698)	0.482	0.255 (−3.155, 5.061)	0.902	0.111 (−0.073, 0.479)	0.174

Boldface indicates statistical significance

Adjusted for sex, age, race, education, marital status, BMI, family income to poverty, alcohol intake daily and smoking. *MVWA* moderate-to-vigorous work activity, *MVRA* moderate-to-vigorous recreational activity

**Table 9:** Subgroup analysis of physical activity, sedentary behavior and NAFLD stratified by MetS

	without MetS		MetS	
	OR (95% CI)	*P*-value	OR (95% CI)	*P*-value
MVWA group				
Insufficient	ref		ref	
Sufficient	1.21(0.69,2.11)	0.48	1.45(0.93,2.27)	0.1
MVRA group				
Insufficient	ref		ref	
Sufficient	0.55(0.35,0.87)	**0.01**	0.68(0.44,1.07)	0.09
Walking/Bicycling group				
Insufficient	ref		ref	
Sufficient	1.27(0.74,2.20)	0.36	1.09(0.49,2.41)	0.82
Sedentary behavior group				
Insufficient	ref		ref	
Sufficient	1.20(0.72,2.01)	0.46	0.91(0.39,2.13)	0.82

Boldface indicates statistical significance

Adjusted for sex, age, race, education, marital status, BMI, family income to poverty, alcohol intake daily and smoking. *MetS* metabolic syndrome, *CI* confidence interval, *MVWA* moderate-to-vigorous work activity, *MVRA* moderate-to-vigorous recreational activity

## Discussion

Among the four physical activity patterns, only recreational activity was associated with a lower risk of NAFLD, and approximately 17.9% of this relationship was mediated by MetS. In individuals without MetS, sufficient recreational activity was linked to a lower risk of NAFLD. Although sedentary behavior was associated with a higher risk of MetS, it did not appear to be related to NAFLD. Additionally, sufficient work activity or walking/bicycling (for commuting) does not appear to have a significant relationship with either NAFLD or MetS.

NAFLD can be attributed to various mechanisms, but the most influential factor is insulin resistance (IR), which is considered the strongest predictor of NAFLD development and is directly linked to metabolic disorders [26]. Insulin resistance, also a central component of metabolic syndrome, along with chronic subclinical inflammation, are regarded as the primary molecular factors underlying the pathogenesis of NAFLD [27]. Within the spectrum of overnutrition, metabolic syndrome, and type 2 diabetes, IR is closely intertwined with ectopic lipid accumulation and could be linked to MetS and NAFLD [28]. This may represent a crucial mechanism through which metabolic syndrome mediates the development of NAFLD.

Numerous studies have demonstrated that exercise is effective in reducing insulin resistance associated with metabolic syndrome [29]. Recent research has also revealed that the lipid status of the liver has a dose-dependent effect on skeletal muscle metabolism, the organ system most visibly affected by physical activity [30]. Approximately 60–80% of glucose metabolism stimulated by insulin occurs in skeletal muscle [31]. Therefore, by enhancing metabolic activity in skeletal muscle and significantly improving insulin resistance associated with metabolic issues, exercise can greatly improve the quality of life for individuals with metabolic syndrome and NAFLD, particularly benefiting cardiometabolic health in overweight or obese adults with respect to blood pressure, insulin resistance, and intrahepatic fat [32]. Exercise not only helps improve insulin resistance and reduce inflammatory responses in individuals with obesity but also enhances immunity and delays the aging of immune cells. Proteomic analysis revealed that consistent exercise training triggers hepatokine secretion remodeling, which alters the metabolism of the liver and skeletal muscle. In mouse models, a novel hepatokine called SDC4 responds to exercise and reduces fatty acid uptake and liver steatosis, offering potential insights into novel targets for NAFLD treatment by elucidating the proteome alterations in hepatocytes due to exercise [33].

We have found that recreational activities have a more significant effect on reducing the risk of NAFLD than do work activities or walking/bicycling (for commuting). This effect may be attributed to the distinct mechanisms by which different types of activities influence physical health and metabolic conditions. Recreational activity, typically characterized by moderate to high-intensity aerobic exercise, are often planned and structured, such as swimming, fitness, and various sports, and can significantly improve insulin resistance, decrease metabolic risk factors, and enhance overall metabolic health [29]. Work activity, on the other hand, may be characterized by prolonged periods of static posture, a lack of control over the duration of activity, and insufficient rest, which may lack health benefits [34]. Walking and cycling, as daily commuting methods, do contribute to physical activity; however, their effectiveness in offering significant health benefits is limited by factors such as the work environment and commuting distance.

The impact of recreational activity versus other types of activities on NAFLD is distinct, a phenomenon referred to as the"physical activity paradox," [35] which may be attributed to various factors. Multiple randomized controlled trials comparing populations with different exercise intensities have found that high-intensity exercise significantly reduces visceral fat content and improves liver function compared to low-intensity exercise [36, 37]. Similarly, our results also demonstrate that vigorous recreational exercise is associated with lower NAFLD. This may be related to the sustained improvement in cardiorespiratory fitness and metabolic levels following high-intensity exercise, which enhances fat breakdown [38]. In terms of exercise purpose, recreational activities are typically chosen and managed with the goal of improving health, accompanied by positive emotional experiences and social interactions. These activities may help reduce stress and, in turn, lower the risk of dyslipidemia. In contrast, work-related and commuting activities are often driven by daily life demands and may be associated with stress or fatigue, which could counteract their potential benefits [39, 40]. Additionally, research indicates that recreational activities can significantly alleviate adverse mental states such as anxiety and depression, which are closely linked to MetS or NAFLD [41–43].

Previous studies have also found that sedentary behavior is significantly linked to an increased risk of obesity, type II diabetes, and NAFLD [44]. Although our study did not establish a definitive link between sedentary behavior and NAFLD, it did reveal a significant association between prolonged sitting and MetS. Sedentary behavior reduces energy expenditure, leading to a positive energy balance and fat accumulation, particularly a noticeable increase in visceral fat [45]. Additionally, with decreased muscle activity, insulin resistance is exacerbated, resulting in an increased risk of metabolic diseases and mortality [46]. The increase in sedentary time simultaneously affects the duration of physical activity, with the average daily steps decreasing by 10,285 steps, leading to an additional 223 min/day of sedentary behavior [47]. This, in turn, results in a reversible decline in insulin sensitivity across multiple organs and cardiorespiratory endurance, accompanied by increased central and hepatic fat accumulation as well as dyslipidemia. Kelly et al. [48] has also indicated that, independent of other levels of physical activity, greater sedentary time is linked to metabolic dysfunction. Switching from a sedentary lifestyle to regular exercise can result in modest improvements in liver enzyme levels, blood lipids, blood glucose, insulin resistance, and BMI [18]. Therefore, in addition to increasing moderate-to-vigorous recreational activities, reducing sedentary time is also a crucial strategy for enhancing metabolic health.

Sufficient recreational activity does not always correlate with a reduced risk of NAFLD. In our study, we did not identify any specific exercise patterns that significantly improved NAFLD in patients with MetS. This finding further supports the notion that MetS may play a mediating role in the relationship between recreational activity and NAFLD. In individuals without MetS, engaging in leisure-time exercise may improve metabolic health, thereby reducing the risk of both MetS and NAFLD. However, once MetS is present, this protective effect seems to be lost, highlighting the potential underlying mechanisms through which MetS impedes the benefits of exercise on NAFLD.

In addition, we observed that certain findings varied between male and female subgroups, suggesting potential sex-specific differences in the associations under investigation. Although recreational activity lowers the CAP index in females, it doesn't consistently reduce NAFLD risk, which is significantly associated with activity in males. Moreover, the mediating effect of metabolic syndrome was no longer significant within the gender subgroups, which may be attributable to various factors. There are significant differences between males and females in terms of metabolism and endocrinology, which may influence the role of MetS as a mediating factor.

Women typically have a lower basal metabolic rate, leading to relatively lower exercise intensity [49]. In contrast, men are more likely to engage in high-intensity recreational activities, which may result in more pronounced reductions in liver fat [50]. Moreover, there are significant gender differences in fat distribution, which may further explain the observed variations. Females tend to store fat in peripheral areas such as the hips and thighs, while males are more likely to exhibit visceral obesity, with visceral fat deposition being more directly linked to MetS and NAFLD [51, 52]. Previous research [53] suggests that exercise training results in a greater proportional decrease in visceral adipose tissue than in subcutaneous adipose tissue among overweight individuals. The differences in fat deposition and metabolism between genders may further explain the findings observed in our subgroups. The protective effect of physical activity on MetS also differs between genders. One study found that the protective effect of physical activity frequency on MetS was linear in women, whereas it was exponential in men [54]. This suggests that men may experience a greater reduction in MetS risk from higher levels of physical activity, which could, in turn, indirectly lower the risk of NAFLD. Sex hormones also have a significant impact on metabolism. Estrogen may offer protective effects against hepatic fat accumulation, while testosterone may increase the risk of liver fat deposition [55]. After menopause, the reduction in estrogen levels may make women more susceptible to developing NAFLD. This complex hormonal change in women could further influence the mediating effect of metabolic syndrome.

Additionally, dietary patterns may also play a role in the development of MetS and NAFLD. The Mediterranean diet, rich in fiber, olive oil, and fish, and low in sugar and processed foods, is often regarded as a model of healthy eating, as it effectively reduces the risk of metabolic syndrome and may even have therapeutic effects on NAFLD [56]. Matteis et al. further combined dietary components with factors such as eating rhythms and physical activity to create a dietary score for effectively identifying individuals with visceral obesity [57]. This dietary pattern also differs between genders, with women generally showing a greater preference for healthy nutrition and being more likely to engage in behaviors such as controlling their weight through diet [58, 59]. These gender-specific dietary behaviors, along with biological factors, may further complicate the mediating role of MetS. Finally, stratified analyses reduce the sample size within each subgroup, which may decrease the statistical power and result in originally present mediation effects becoming statistically non-significant.

### Limitations

There are also limitations in our study. Firstly, the cross-sectional design precludes causal inference. Future longitudinal studies should prospectively track activity patterns, MetS development, and NAFLD incidence. For example, cohorts with repeated measures could assess whether recreational activity changes influence MetS occurrence and thereby modulate NAFLD risk, controlling for baseline confounders. Secondly, the self-reported physical activity data introduce potential misclassification bias. Objective measures (e.g., accelerometry-derived activity profiles) are needed to distinguish domain-specific effects (e.g., occupational vs. leisure activity). Thirdly, while we adjusted for BMI, dietary patterns (e.g., macronutrient composition) were insufficiently captured. Subsequent investigations should incorporate validated dietary assessment tools (e.g., 24-h dietary recalls) to examine effect modification by macronutrient composition. For example, a randomized controlled trial (RCT) could test whether combining physical activity interventions with dietary modifications (e.g., Mediterranean diet protocols) attenuates MetS-mediated pathological effects on NAFLD development. Finally, the observed sex-specific heterogeneity in MetS mediation (e.g., nonsignificant associations in females) requires confirmation in larger cohorts. A stratified longitudinal analysis could clarify whether biological (e.g., hormonal variations) factors drive these disparities.

## Conclusion

Our study revealed that approximately 18% of the association between sufficient recreational activity and liver steatosis may be mediated by MetS, whereas no mediating role was identified for sedentary behavior. It is strongly urged that sufficient RA may significantly reduce the risk of hepatic steatosis by cutting off the mediation of MetS and providing additional health benefits. Additionally, sedentary behavior is correlated with an increased risk of MetS.

## Supplementary Information

Supplementary Material 1.

## Data Availability

The datasets analysed during the current study are available in the NHANES repository, https://wwwn.cdc.gov/nchs/nhanes/Default.aspx.

## Abbreviations

NAFLD: Non-alcoholic fatty liver disease
HCC: Hepatocellular carcinoma
TE: Transient elastography
CAP: Controlled-attenuation parameter
LSM: Liver stiffness measurement
MetS: Metabolic syndrome
MAFLD: Metabolic dysfunction-associated fatty liver disease
PA: Physical activity
SB: Sedentary behavior
NHANES: National Health and Nutrition Examination Surveys
GPAQ: Global Physical Activity Questionnaire
WHO: World Health Organization
MPA: Moderate-intensity aerobic physical activity
VPA: Vigorous-intensity aerobic physical activity
MVWA: Moderate-to-vigorous work activity
MVRA: Moderate-to-vigorous recreational activity
WC: Waist circumference
BMI: Body mass index
TG: Triglycerides
FPG: Fasting plasma glucose
CI: Confidence intervals
ALT: Alanine aminotransferase
ALP: Alkaline phosphatase
AST: Aspartate aminotransferase
GGT: Gamma-glutamyl transferase
LDH: Lactate dehydrogenase
IR: Insulin resistance

## References

[1] Le MH, Yeo YH, Li X, et al. 2019 Global NAFLD prevalence: a systematic review and meta-analysis. Clin Gastroenterol Hepatol. 2022;20:2809-2817.e28. 34890795 10.1016/j.cgh.2021.12.002

[2] Quek J, Chan KE, Wong ZY, et al. Global prevalence of non-alcoholic fatty liver disease and non-alcoholic steatohepatitis in the overweight and obese population: a systematic review and meta-analysis. Lancet Gastroenterol Hepatol. 2023;8:20–30. 36400097 10.1016/S2468-1253(22)00317-X

[3] Estes C, Razavi H, Loomba R, Younossi Z, Sanyal AJ. Modeling the epidemic of nonalcoholic fatty liver disease demonstrates an exponential increase in burden of disease. Hepatology. 2017;67:123–33. 28802062 10.1002/hep.29466 PMC5767767

[4] Choi S, Kim BK, Yon DK, et al. Global burden of primary liver cancer and its association with underlying aetiologies, sociodemographic status, and sex differences from 1990–2019: a DALY-based analysis of the Global Burden of Disease 2019 study. Clin Mol Hepatol. 2023;29:433–52. 36597018 10.3350/cmh.2022.0316 PMC10121317

[5] Cardoso CRL, Villela-Nogueira CA, Leite NC, Salles GF. Prognostic impact of liver fibrosis and steatosis by transient elastography for cardiovascular and mortality outcomes in individuals with nonalcoholic fatty liver disease and type 2 diabetes: the Rio de Janeiro Cohort Study. Cardiovasc Diabetol. 2021;20:193. 34560854 10.1186/s12933-021-01388-2 PMC8464106

[6] Vilar-Gomez E, Vuppalanchi R, Gawrieh S, Samala N, Chalasani N. CAP and LSM as determined by VCTE are independent predictors of all-cause mortality in the US adult population. Hepatology. 2023;77:1241–52. 36626638 10.1097/HEP.0000000000000023

[7] Hirode G, Wong RJ. Trends in the prevalence of metabolic syndrome in the United States, 2011–2016. JAMA. 2020;323:2526. 32573660 10.1001/jama.2020.4501 PMC7312413

[8] Lonardo A, Mantovani A, Lugari S, Targher G. NAFLD in some common endocrine diseases: prevalence, pathophysiology, and principles of diagnosis and management. Int J Mol Sci. 2019;20:2841. 31212642 10.3390/ijms20112841 PMC6600657

[9] Kneeman JM, Misdraji J, Corey KE. Secondary causes of nonalcoholic fatty liver disease. Ther Adv Gastroenterol. 2011;5:199–207. 10.1177/1756283X11430859 PMC3342568 22570680

[10] Carvalho-Gontijo R, Han C, Zhang L, et al. Metabolic injury of hepatocytes promotes progression of NAFLD and AALD. Semin Liver Dis. 2022;42:233–49. 36001995 10.1055/s-0042-1755316 PMC9662188

[11] Stepanova M, Rafiq N, Younossi ZM. Components of metabolic syndrome are independent predictors of mortality in patients with chronic liver disease: a population-based study. Gut. 2010;59:1410–5. 20660697 10.1136/gut.2010.213553

[12] Diao Y, Tang J, Wang X, Deng W, Tang J, You C. Metabolic syndrome, nonalcoholic fatty liver disease, and chronic hepatitis B: a narrative review. Infect Dis Ther. 2022;12:53–66. 36441483 10.1007/s40121-022-00725-6 PMC9868033

[13] Eslam M, Newsome PN, Sarin SK, et al. A new definition for metabolic dysfunction-associated fatty liver disease: an international expert consensus statement. J Hepatol. 2020;73:202–9. 32278004 10.1016/j.jhep.2020.03.039

[14] Han Q, Han X, Wang X, et al. Association of accelerometer-measured sedentary behavior patterns with nonalcoholic fatty liver disease among older adults: the MIND-China study. Am J Gastroenterol. 2022;118:569–73. 36621973 10.14309/ajg.0000000000002135

[15] Wu J, Zhang H, Yang L, et al. Sedentary time and the risk of metabolic syndrome: a systematic review and dose–response meta-analysis. Obes Rev. 2022;23:e13510. 36261077 10.1111/obr.13510

[16] Von Loeffelholz C, Roth J, Coldewey S, Birkenfeld A. The role of physical activity in nonalcoholic and metabolic dysfunction associated fatty liver disease. Biomedicines. 2021;9:1853. 34944668 10.3390/biomedicines9121853 PMC8698784

[17] Vilar-Gomez E, Nephew LD, Vuppalanchi R, Gawrieh S, Mladenovic A, Pike F, Samala N, Chalasani N. High-quality diet, physical activity, and college education are associated with low risk of NAFLD among the US population. Hepatology. 2021;75:1491–506. 34668597 10.1002/hep.32207

[18] Ma Q, Ye J, Shao C, Lin Y, Wu T, Zhong B. Metabolic benefits of changing sedentary lifestyles in nonalcoholic fatty liver disease: a meta-analysis of randomized controlled trials. Ther Adv Endocrinol Metab. 2022;13:20420188221122424. 36147997 10.1177/20420188221122426 PMC9486298

[19] Yang L, Cao C, Kantor ED, Nguyen LH, Zheng X, Park Y, Giovannucci EL, Matthews CE, Colditz GA, Cao Y. Trends in sedentary behavior among the US population, 2001–2016. JAMA. 2019;321:1587. 31012934 10.1001/jama.2019.3636 PMC6487546

[20] Siddiqui MS, Vuppalanchi R, Van Natta ML, et al. Vibration-controlled transient elastography to assess fibrosis and steatosis in patients with nonalcoholic fatty liver disease. Clin Gastroenterol Hepatol. 2019;17:156-163.e2. 29705261 10.1016/j.cgh.2018.04.043 PMC6203668

[21] Sookoian S, Pirola CJ. The serum uric acid/creatinine ratio is associated with nonalcoholic fatty liver disease in the general population. J Physiol Biochem. 2022;79:891–9. 35546386 10.1007/s13105-022-00893-6

[22] Cleland CL, Hunter RF, Kee F, Cupples ME, Sallis JF, Tully MA. Validity of the Global Physical Activity Questionnaire (GPAQ) in assessing levels and change in moderate-vigorous physical activity and sedentary behaviour. BMC Public Health. 2014;14:1255. 25492375 10.1186/1471-2458-14-1255 PMC4295403

[23] Bull FC, Al-Ansari SS, Biddle S, et al. World Health Organization 2020 guidelines on physical activity and sedentary behaviour. Br J Sports Med. 2020;54:1451–62. 33239350 10.1136/bjsports-2020-102955 PMC7719906

[24] Huang B, Huang Z, Tan J, Xu H, Deng K, Cheng J, Ren Z, Gong X, Gao Y. The mediating and interacting role of physical activity and sedentary behavior between diabetes and depression in people with obesity in United States. J Diabetes Complications. 2021;35:107764. 33616042 10.1016/j.jdiacomp.2020.107764

[25] Alberti KGMM, Eckel RH, Grundy SM, Zimmet PZ, Cleeman JI, Donato KA, Fruchart J-C, James WPT, Loria CM, Smith SC. Harmonizing the metabolic syndrome: a joint interim statement of the international diabetes federation task force on epidemiology and prevention; National Heart, Lung, and Blood Institute; American Heart Association; World Heart Federation; International Atherosclerosis Society; and International Association for the Study of Obesity. Circulation. 2009;120:1640–5. 19805654 10.1161/CIRCULATIONAHA.109.192644

[26] Stefan N, Häring HU, Cusi K. Non-alcoholic fatty liver disease: causes, diagnosis, cardiometabolic consequences, and treatment strategies. Lancet Diabetes Endocrinol. 2019;7:313–24. 30174213 10.1016/S2213-8587(18)30154-2

[27] Tilg H, Moschen AR, Roden M. NAFLD and diabetes mellitus. Nat Rev Gastroenterol Hepatol. 2016;14:32–42. 27729660 10.1038/nrgastro.2016.147

[28] Von Loeffelholz C, Coldewey SM, Birkenfeld AL. A narrative review on the role of AMPK on de novo lipogenesis in non-alcoholic fatty liver disease: evidence from human studies. Cells. 2021;10:1822. 34359991 10.3390/cells10071822 PMC8306246

[29] Roberts CK, Hevener AL, Barnard RJ. Metabolic syndrome and insulin resistance: underlying causes and modification by exercise training. Compr Physiol. 2013;3(1):1–58. 10.1002/cphy.c110062. 10.1002/cphy.c110062 PMC4129661 23720280

[30] Bril F, Barb D, Portillo-Sanchez P, Biernacki D, Lomonaco R, Suman A, Weber MH, Budd JT, Lupi ME, Cusi K. Metabolic and histological implications of intrahepatic triglyceride content in nonalcoholic fatty liver disease. Hepatology. 2017;65:1132–44. 27981615 10.1002/hep.28985

[31] Ng JM, Azuma K, Kelley C, Pencek R, Radikova Z, Laymon C, Price J, Goodpaster BH, Kelley DE. PET imaging reveals distinctive roles for different regional adipose tissue depots in systemic glucose metabolism in nonobese humans. Am J Physiol-Endocrinol Metab. 2012;303:E1134–41. 22967498 10.1152/ajpendo.00282.2012 PMC3492855

[32] Battista F, Ermolao A, Van Baak MA, et al. Effect of exercise on cardiometabolic health of adults with overweight or obesity: focus on blood pressure, insulin resistance, and intrahepatic fat—a systematic review and meta-analysis. Obes Rev. 2021;22:e13269. 33960110 10.1111/obr.13269 PMC8365642

[33] De Nardo W, Miotto PM, Bayliss J, Nie S, Keenan SN, Montgomery MK, Watt MJ. Proteomic analysis reveals exercise training induced remodelling of hepatokine secretion and uncovers syndecan-4 as a regulator of hepatic lipid metabolism. Mol Metab. 2022;60:101491. 35381388 10.1016/j.molmet.2022.101491 PMC9034320

[34] Cuthbertson CC, Moore CC, Evenson KR. Paradox of occupational and leisure-time physical activity associations with cardiovascular disease. Heart. 2023;109:656–8. 10.1136/heartjnl-2022-321856 PMC10314960 36593098

[35] Bonekamp NE, Visseren FLJ, Ruigrok Y, Cramer MJM, de Borst GJ, May AM, Koopal C, UCC-SMART Study group, UCC-SMART study group. Leisure-time and occupational physical activity and health outcomes in cardiovascular disease. Heart. 2022;109:686–94. 10.1136/heartjnl-2022-321474 36270785

[36] Zhang HJ, He J, Pan LL, et al. Effects of moderate and vigorous exercise on nonalcoholic fatty liver disease: a randomized clinical trial. JAMA Intern Med. 2016;176:1074. 27379904 10.1001/jamainternmed.2016.3202

[37] Irving BA, Davis CK, Brock DW, Weltman JY, Swift D, Barrett EJ, Gaesser GA, Weltman A. Effect of exercise training intensity on abdominal visceral fat and body composition. Med Sci Sports Exerc. 2008;40:1863–72. 18845966 10.1249/MSS.0b013e3181801d40 PMC2730190

[38] Yoshioka M, Doucet E, St-Pierre S, Alméras N, Richard D, Labrie A, Després JP, Bouchard C, Tremblay A. Impact of high-intensity exercise on energy expenditure, lipid oxidation and body fatness. Int J Obes. 2001;25:332–9. 10.1038/sj.ijo.0801554 11319629

[39] Assadi SN. What are the effects of psychological stress and physical work on blood lipid profiles? Medicine (Baltimore). 2017;96:e6816. 28471984 10.1097/MD.0000000000006816 PMC5419930

[40] Tsai SY. A study of the health-related quality of life and work-related stress of white-collar migrant workers. Int J Environ Res Public Health. 2012;9:3740–54. 23202771 10.3390/ijerph9103740 PMC3509477

[41] Wheeler M, Cooper NR, Andrews L, Hacker Hughes J, Juanchich M, Rakow T, Orbell S. Outdoor recreational activity experiences improve psychological wellbeing of military veterans with post-traumatic stress disorder: positive findings from a pilot study and a randomised controlled trial. PLoS ONE. 2020;15:e0241763. 33237906 10.1371/journal.pone.0241763 PMC7688151

[42] Hoffmann MS, Brunoni AR, Stringaris A, Viana MC, Lotufo PA, Benseñor IM, Salum GA. Common and specific aspects of anxiety and depression and the metabolic syndrome. J Psychiatr Res. 2021;137:117–25. 33677215 10.1016/j.jpsychires.2021.02.052

[43] Shea S, Lionis C, Kite C, Atkinson L, Chaggar SS, Randeva HS, Kyrou I. Non-Alcoholic Fatty Liver Disease (NAFLD) and potential links to depression, anxiety, and chronic stress. Biomedicines. 2021;9:1697. 34829926 10.3390/biomedicines9111697 PMC8615558

[44] Bishop NC, Wadley AJ, Hamrouni M, Roberts MJ. Inactivity and obesity: consequences for macrophage-mediated inflammation and the development of cardiometabolic disease. Proc Nutr Soc. 2022;82:13–21. 35996926 10.1017/S0029665122002671

[45] Hamilton MT, Hamilton DG, Zderic TW. Role of low energy expenditure and sitting in obesity, metabolic syndrome, type 2 diabetes, and cardiovascular disease. Diabetes. 2007;56:2655–67. 17827399 10.2337/db07-0882

[46] Bergouignan A, Rudwill F, Simon C, Blanc S. Physical inactivity as the culprit of metabolic inflexibility: evidence from bed-rest studies. J Appl Physiol. 2011;111:1201–10. 21836047 10.1152/japplphysiol.00698.2011

[47] Bowden Davies KA, Sprung VS, Norman JA, Thompson A, Mitchell KL, Halford JCG, Harrold JA, Wilding JPH, Kemp GJ, Cuthbertson DJ. Short-term decreased physical activity with increased sedentary behaviour causes metabolic derangements and altered body composition: effects in individuals with and without a first-degree relative with type 2 diabetes. Diabetologia. 2018;61:1282–94. 29671031 10.1007/s00125-018-4603-5

[48] Bowden Davies KA, Sprung VS, Norman JA, et al. Physical activity and sedentary time: association with metabolic health and liver fat. Med Sci Sports Exerc. 2019;51:1169–77. 30694971 10.1249/MSS.0000000000001901 PMC6542688

[49] Verma N, Kumar SS, Suresh A. An evaluation of basal metabolic rate among healthy individuals — a cross-sectional study. Bull Fac Phys Ther. 2023;28:26.

[50] Kretschmer L, Salali GD, Andersen LB, Hallal PC, Northstone K, Sardinha LB, Dyble M, Bann D, International Children’s Accelerometry Database (ICAD) Collaborators. Gender differences in the distribution of children’s physical activity: evidence from nine countries. Int J Behav Nutr Phys Act. 2023;20:103. 37667391 10.1186/s12966-023-01496-0 PMC10478357

[51] Kolnes KJ, Petersen MH, Lien-Iversen T, Højlund K, Jensen J. Effect of exercise training on fat loss—energetic perspectives and the role of improved adipose tissue function and body fat distribution. Front Physiol. 2021;12:737709. 34630157 10.3389/fphys.2021.737709 PMC8497689

[52] Pradhan AD. Sex differences in the metabolic syndrome: implications for cardiovascular health in women. Clin Chem. 2014;60:44–52. 24255079 10.1373/clinchem.2013.202549

[53] Langleite TM, Jensen J, Norheim F, et al. Insulin sensitivity, body composition and adipose depots following 12 w combined endurance and strength training in dysglycemic and normoglycemic sedentary men. Arch Physiol Biochem. 2016;122:167–79. 27477619 10.1080/13813455.2016.1202985

[54] Chen SP, Chang HC, Hsiao TM, Yeh CJ, Yang HJ. Gender differences in the effects of the frequency of physical activity on the incidence of metabolic syndrome: results from a middle-aged community cohort in Taiwan. Metab Syndr Relat Disord. 2018;16:224–31. 29688799 10.1089/met.2017.0154

[55] Ballestri S, Nascimbeni F, Baldelli E, Marrazzo A, Romagnoli D, Lonardo A. NAFLD as a sexual dimorphic disease: role of gender and reproductive status in the development and progression of nonalcoholic fatty liver disease and inherent cardiovascular risk. Adv Ther. 2017;34:1291–326. 28526997 10.1007/s12325-017-0556-1 PMC5487879

[56] Anania C, Perla FM, Olivero F, Pacifico L, Chiesa C. Mediterranean diet and nonalcoholic fatty liver disease. World J Gastroenterol. 2018;24:2083–94. 29785077 10.3748/wjg.v24.i19.2083 PMC5960814

[57] De Matteis C, Crudele L, Battaglia S, et al. Identification of a novel score for adherence to the mediterranean diet that is inversely associated with visceral adiposity and cardiovascular risk: the Chrono Med Diet Score (CMDS). Nutrients. 2023;15:1910. 37111129 10.3390/nu15081910 PMC10141687

[58] Grzymisławska M, Puch EA, Zawada A, Grzymisławski M. Do nutritional behaviors depend on biological sex and cultural gender? Adv Clin Exp Med. 2020;29:165–72. 32017478 10.17219/acem/111817

[59] Pant A, Chew DP, Mamas MA, Zaman S. Cardiovascular disease and the mediterranean diet: insights into sex-specific responses. Nutrients. 2024;16:570. 38398894 10.3390/nu16040570 PMC10893368

