# Supplementary material for: Mediational role of metabolic syndrome between physical activity, sedentary behavior and non-alcoholic fatty liver disease: a cross-sectional study
Source: BMC Public Health. 2025 May 6;25:1661. doi: 10.1186/s12889-025-22925-8 (PMC12054284; doi:10.1186/s12889-025-22925-8)
Supplement: Supplementary file 1 — Supplementary Material 1. [file 12889_2025_22925_MOESM1_ESM.docx]

Supplementary Table S1 Physical activity questionnaire from the NHANES

| **Physical Activity** | | |
| --- | --- | --- |
| Next I am going to ask you about the time you spend doing different types of physical activity in a typical week. | | |
| **Activity at work** | Think first about the time you spend doing work.Think of work as the things that you have to do such as paid or unpaid work, study/training, household chores, harvesting food/crops, fishing or hunting for food, seeking employment.In answering the following questions 'vigorous-intensity activities' are activities that require hard physical effort and cause large increases in breathing or heart rate, 'moderate-intensity activities' are activities that require moderate physical effort and cause small increases in breathing or heart rate. | |
| VWA  (vigorous-intensity activity) | PAQ605 | does {your/sp's} work involve vigorous-intensity activity that causes large increases in breathing or heart rate like carrying or lifting heavy loads, digging or construction work for at least 10 minutes continuously? |
|  | PAQ610 | in a typical week, on how many days {do you/does sp} do vigorous-intensity activities as part of {your/his/her} work? |
|  | PAD615 | how much time {do you/does sp} spend doing vigorous-intensity activities at work on a typical day? |
| MWA  (moderate-intensity work activity) | PAQ620 | does {your/sp's} work involve moderate-intensity activity that causes small increases in breathing or heart rate such as brisk walking or carrying light loads for at least 10 minutes continuously? |
|  | PAQ625 | in a typical week, on how many days {do you/does sp} do moderate-intensity activities as part of {your/his/her} work? |
|  | PAD630 | how much time {do you/does sp} spend doing moderate-intensity activities at work on a typical day? |
| **Travel to and from places** | The next questions exclude the physical activities at work that you have already mentioned. | |
| Walking/Bicycling | PAQ635 | now i would like to ask you about the usual way {you travel/sp travels} to and from places. for example to school, for shopping, to work. in a typical week {do you/does sp} walk or use a bicycle for at least 10 minutes continuously to get to and from places? |
|  | PAQ640 | in a typical week, on how many days {do you/does sp} walk or bicycle for at least 10 minutes continuously to get to and from places? |
|  | PAD645 | how much time {do you/does sp} spend walking or bicycling for travel on a typical day? |
| **Recreational activities** | The next questions exclude the work and transport activities that you have already mentioned. Now I would like to ask you about sports, fitness and recreational activities (leisure) | |
| VRA  (vigorous- recreational activity) | PAQ650 | now i would like to ask you about sports, fitness and recreational activities. in a typical week {do you/does sp} do any vigorous-intensity sports, fitness, or recreational activities that cause large increases in breathing or heart rate like running or basketball for at least 10 minutes continuously? |
|  | PAQ655 | in a typical week, on how many days {do you/does sp} do vigorous-intensity sports, fitness or recreational activities? |
|  | PAD660 | how much time {do you/does sp} spend doing vigorous-intensity sports, fitness or recreational activities on a typical day? |
| MRA  (moderate- recreational activity) | PAQ665 | in a typical week {do you/does sp} do any moderate-intensity sports, fitness, or recreational activities that cause a small increase in breathing or heart rate such as brisk walking, bicycling, swimming, or volleyball for at least 10 minutes continuously? |
|  | PAQ670 | in a typical week, on how many days {do you/does sp} do moderate-intensity sports, fitness or recreational activities? |
|  | PAD675 | how much time {do you/does sp} spend doing moderate-intensity sports, fitness or recreational activities on a typical day? |
| **Sedentary behavior** | | |
| **Sedentary behavior** | PAD680 | the following question is about sitting at school, at home, getting to and from places, or with friends including time spent sitting at a desk, traveling in a car or bus, reading, playing cards, watching television, or using a computer. do not include time spent sleeping. how much time {do you/does sp} usually spend sitting on a typical day? |

Supplementary Table S2

The interaction effect of physical activity and MetS

| Exposure | MetS | OR (95%CI) | P-value | P for interaction |  | β (95%CI) | P-value | P for interaction |
| --- | --- | --- | --- | --- | --- | --- | --- | --- |
| MVWA group |  |  |  |  |  |  |  |  |
| Insufficient | no | Ref. |  | 0.8194 |  | Ref. |  | 0.6355 |
| Sufficient | no | 1.1 (0.8, 1.4) | 0.6513 |  |  | 0.4 (-4.4, 5.2) | 0.8647 |  |
| Insufficient | yes | 2.6 (2.1, 3.3) | <0.0001 |  |  | 25.7 (20.8, 30.6) | <0.0001 |  |
| Sufficient | yes | 2.7 (2.0, 3.5) | <0.0001 |  |  | 27.9 (22.2, 33.6) | <0.0001 |  |
| MVRA group |  |  |  |  |  |  |  |  |
| Insufficient | no | Ref. |  | 0.5569 |  | Ref. |  | 0.6092 |
| Sufficient | no | 0.8 (0.6, 1.0) | 0.0496 |  |  | -7.6 (-12.4, -2.7) | 0.0024 |  |
| Insufficient | yes | 2.6 (2.1, 3.3) | <0.0001 |  |  | 26.6 (22.0, 31.1) | <0.0001 |  |
| Sufficient | yes | 1.8 (1.4, 2.4) | <0.0001 |  |  | 17.0 (10.8, 23.2) | <0.0001 |  |
| Walking/bicycling group |  |  |  |  |  |  |  |  |
| Insufficient | no | Ref. |  | **0.0389** |  | Ref. |  | 0.3499 |
| Sufficient | no | 1.2 (0.8, 1.7) | 0.4054 |  |  | -0.0 (-6.9, 6.8) | 0.9938 |  |
| Insufficient | yes | 2.7 (2.3, 3.3) | <0.0001 |  |  | 26.9 (22.8, 31.1) | <0.0001 |  |
| Sufficient | yes | 1.8 (1.2, 2.7) | 0.0037 |  |  | 21.6 (12.7, 30.5) | <0.0001 |  |
| Sedentary behavior group |  |  |  |  |  |  |  |  |
| no | no | Ref. |  | 0.3183 |  | Ref. |  | 0.7334 |
| yes | no | 1.3 (1.0, 1.7) | 0.0583 |  |  | 0.0 (-5.2, 5.3) | 0.9921 |  |
| no | yes | 2.7 (2.2, 3.4) | <0.0001 |  |  | 26.0 (21.4, 30.5) | <0.0001 |  |
| yes | yes | 2.9 (2.2, 3.9) | <0.0001 |  |  | 27.3 (21.4, 33.3) | <0.0001 |  |

Adjusted for sex, age, race, education, marital status, BMI, family income to poverty, alcohol intake daily and smoking. MetS: metabolic syndrome; OR: effect value when outcomes are categorical variables; CI, confidence interval; β: effect value when outcomes are continuous variables. MVWA: moderate-to-vigorous work activity; MVRA: moderate-to-vigorous recreational activity.
